# Health Concerns and Health Service Utilization in a Population Cohort of Young Adults with Autism Spectrum Disorder

**DOI:** 10.1007/s10803-017-3292-0

**Published:** 2017-09-13

**Authors:** Jonathan A. Weiss, Barry Isaacs, Heidi Diepstra, Andrew S. Wilton, Hilary K. Brown, Caitlin McGarry, Yona Lunsky

**Affiliations:** 1 Department of Psychology, York University, 4700 Keele St., 230 Behavioural Sciences Building, Toronto, ON M3J 1P3 Canada; 2 Surrey Place Centre, Toronto, ON Canada; 3 Institute for Clinical Evaluative Sciences, Toronto, ON Canada; 4 Department of Health Studies, University of Toronto Scarborough, Scarborough, ON Canada; 5 Centre for Addiction and Mental Health, Toronto, ON Canada; 6 University of Toronto, Toronto, ON Canada; 7 Cancer Care Ontario, Toronto, ON Canada

**Keywords:** Autism, Developmental disability, Health, Services, Epidemiology, Psychiatry, Comorbidity

## Abstract

Individuals with autism spectrum disorder (ASD) have many health needs that place demands on the health service sector. This study used administrative data to compare health profiles in young adults 18–24 years of age with ASD to peers with and without other developmental disability. Young adults with ASD were more likely to have almost all the examined clinical health issues and health service use indicators compared to peers without developmental disability. They were more likely to have at least one psychiatric diagnosis, and visit the family physician, pediatrician, psychiatrist, and emergency department for psychiatric reasons, compared to peers with other developmental disability. Planning for the mental health care of transition age adults with ASD is an important priority for health policy.

## Introduction

Individuals with autism spectrum disorder (ASD) have core deficits in socio-communicative ability and behavior (American Psychiatric Association 2013). In addition, they experience associated difficulties with health, mental health and challenging behaviors, which can lead to increased service use. With the increasing prevalence of school-aged youth with ASD in recent years, now estimated at 1 in 68 8-year-olds (Centers for Disease Control and Prevention 2014), many are warning of the impending heightened demand for adult services (Gerhardt and Lainer 2011). Service needs will span across sectors, and invariably involve health care. To adequately address future demands, a better understanding of the health needs and health services utilization patterns of young adults with ASD is required. This study used administrative health data to document the profile of health needs of young adults with ASD and compare it to that of typically developing peers and those with other forms of developmental disability (DD).

Previous research indicates that individuals with ASD, across all ages, are at risk for health problems. A recent study by the National Institutes of Mental Health compared rates of health problems in 8325 children and youth with ASD, 3–17 years of age, to 83,195 controls without ASD, based on age, gender, months of enrollment in the health care system, having a prescription drug plan, and the type of health care system they used (Cummings et al. 2016). Patients with ASD were more likely to have a variety of chronic medical conditions compared to controls, including allergies, epilepsy, and neurological, sleep, autoimmune, and endocrine disorders. They were also more likely to have received a psychiatric diagnosis of attention deficit-hyperactivity disorder, oppositional defiant disorder/conduct disorder, anxiety, depression, or other serious mental illness, although less likely to have a substance abuse disorder. In one of the only large scale studies of health conditions specifically for adults with ASD, Croen et al. (2015) reported higher rates of medical conditions (including gastrointestinal problems, sleep issues, immune conditions, seizure disorder, hypertension, and diabetes) and psychiatric disorders in 1507 U.S. adults with ASD aged 18–65 years compared to a sex- and age-matched comparison group of patients without ASD.

In line with this heightened comorbidity, individuals with ASD have higher levels of health service use (Tregnago and Cheak-Zamora 2012). For example, Cummings et al. (2016) reported that children with ASD were more likely than children without ASD to have at least one pediatric visit and more overall visits to the pediatrician, as well as specialty care visits, including speech therapy, occupational or social skills therapy, physical therapy, psychotherapy, and neurology, within a 1 year period. They were also more likely to be hospitalized, have at least one emergency department (ED) visit, and be involved as an outpatient in hospital care. Higher rates of psychiatric-related visits to the ED and hospital admission occur specifically in preadolescents (9–12 years of age) and adolescents (12–18 years of age) with ASD compared to controls (Schlenz et al. 2015). Higher rates of ED visits for psychiatric and non-psychiatric reasons are similarly found for adults with ASD when compared to the general population (Vohra et al. 2016). Children with ASD also use more services, and have greater unmet needs (including for therapeutic, health care, and family support services), compared to children with other special health care needs (Benevides et al. 2016; Kogan et al. 2008). Even when compared to other children with special health care needs who have emotional, developmental, or behavioral problems, children with ASD are reported to have more unmet needs for specialty and therapy care (Chiri and Warfield 2012). Individuals with ASD often experience many barriers to service receipt across the lifespan, as a result of waitlists, a lack of resources, and inadequate service provider skills (Lai and Weiss 2017).

Understanding patterns of need and service use specifically in young adults with ASD is important, given that they are in the transition period between child and adult health care and educational systems (Carbone et al. 2010; Cooley and Sagerman 2011; McDermott et al. 2015), a period in life often reported to be fraught with crisis and service gaps (Cheak-Zamora et al. 2014). Post-high school age youth with ASD are noted to experience a large drop in the use of mental health, medical, speech therapy and case management services when compared to when still in the high school years (Shattuck et al. 2011). Adults with ASD are reported to have greater unmet need across multiple types of services than children and youth with ASD (Lai and Weiss 2017; Turcotte et al. 2016). While we know about medical profiles and service use of children with ASD, and there is an emerging literature on adults with ASD, there is gap in terms of population-based information about young adults (Hamdani and Lunsky 2016).

There is one additional limitation to the reviewed literature. While studies paint a consistent picture of high medical and mental health need, and high rates of health care utilization in individuals with ASD, relative to the general population, this kind of comparison does not consider whether the comorbidities in ASD are indeed unique to this population, or more endemic of DD generally. Individuals with DD without ASD also have greater levels of comorbidities and health service use compared to children without such disabilities (Boulet et al. 2009). If heightened rates are indeed unique to ASD, this would speak to the need for tailored services and health care policies for this population. If these rates are common across individuals with many forms of DD, this would indicate a need for attention to health care policies for a broader array of people with developmental concerns, an even larger group than those with ASD alone. A comparison to peers without DD can provide a reflection for what might be expected based on chronological age alone, but a DD comparison group can provide a benchmark of what might be expected by other individuals with enduring adaptive and cognitive limitations that are also impacted by transition periods (Beresford 2004).

Few large-scale studies exist comparing rates of health need and health service use in individuals with ASD to other DD. Using a population-based register of children up to 18 years of age, including those with intellectual disability (ID), those with ASD (with and without ID), and those with neither condition in Western Australia, Bebbington et al. (2013) found that while rates of hospitalization were higher in those with ASD than the general population, they were not higher than patients with ID only. In contrast, Nayfack et al. (2013) compared rates of hospital discharge in children 1–18 years of age with ASD to rates in children with cerebral palsy, Down syndrome, and to the broader ID category, through a retrospective analysis of hospitalizations during a 10-year period (1999–2009) in California. While there were no changes in the annual rate of discharge for cerebral palsy, Down syndrome, or the general population, rates increased every year for children with ASD. The rise in hospitalizations for children with ASD were most pronounced for children who did not have ID, and was associated with the presence of mental health conditions.

To our knowledge, only one study has examined how patterns of health service use may differ in young adults with ASD compared to peers with and without DD using population-based data, and speaks to the importance of questioning whether patterns of health need and health service use are specific to ASD or more a factor of broader developmental needs. McDermott and colleagues (2015) compared rates of hospital encounters in adolescents and young adults with fragile X syndrome, ASD, and other ID to a non-affected comparison group in South Carolina. Young adults in all three affected groups were more likely to have an inpatient admission, and those with ASD and with ID were more likely to visit the ED, compared to the comparison group. All three DD groups had higher likelihood for hospital admission because of mental illness, a neurological condition, epilepsy/seizures, and pneumonia than those without DD. Those with ASD and with other ID also had a greater likelihood of admission because of a respiratory or genitourinary condition. Although this study included different DD groupings, they failed to compare these groups to one another. Also, their study of health services focused only on ED and inpatient based hospital care, and not outpatient health care use.

The current study aimed to compare the patterns of health needs and health service use in young adults with ASD compared to both young adults with other DD and to the general population. We hypothesized that young adults with ASD would have higher rates of common chronic medical conditions and psychiatric diagnoses and greater levels of primary care, specialized care, emergency care, and hospitalizations compared to young adults with other DD and to young adults without any DD.

## Methods

The demographics, clinical profiles, and health service use patterns of young adults aged 18–24 years with ASD, with other DD (other DD), and those without ASD or other DD (non-DD) in Ontario, Canada were studied using administrative health data. The upper age limit of 24 was chosen through consultation with government policy staff from the children’s, social service and education ministries in Ontario. In Ontario, the switch from child to adult social, and mental services occurs at 18, and individuals with DD typically remain in school until 22. Using the 18–24 age range provides an opportunity to study this group after they age out of child services and are in the process of leaving school. The samples of young adults with ASD and other DD were drawn from a larger cohort of 66,484 adults with DD, aged 18–64 years as of April 1, 2009, living in Ontario (Health Care Access Research and Developmental Disabilities; H-CARDD). This larger cohort of adults with DD was identified by a person-level data linkage of health administrative data (including inpatient, ED, and physician contacts) with Ontario disability income supports from the 2009 fiscal year and contains both people with ASD and other DD. These datasets were linked using unique encoded identifiers and analyzed at the Institute for Clinical Evaluative Sciences (ICES). The data sources, linkage process and the methods for identifying the population with DD are described in detail in prior publications (Lin et al. 2014; Lunsky et al. 2013). Out of the larger cohort, we identified all individuals aged 18–24 years (n = 15,980) and applied an algorithm to identify those with ASD within this subset (see Table 1). A person was categorized as having ASD if they met any one of the following criteria:

- one or more hospital admissions or ED visits with an ASD diagnostic code;
- two or more physician visits with an ASD diagnostic code; or
- an ASD diagnostic code recorded in their disability income support program diagnostic information.

**Table 1:** Diagnostic codes for autism spectrum disorder

Diagnostic System	Code	Label/description
ICD-9	299-299.99	Pervasive developmental disorders (e.g., autism)
ICD-10	F840	Childhood autism
	F841	Atypical autism
	F843	Other childhood disintegrative disorder
	F844	Overactive disorder associated with mental retardation and stereotyped movements
	F845	Asperger’s syndrome
	F848	Other pervasive developmental disorders
	F849	Pervasive developmental disorder, unspecified

Similar to the logic applied in developing the larger cohort of individuals with DD (Lin et al. 2012), two instances were required in the physician visits criterion. Using two physician visit codes or one from another source has good specificity and moderate sensitivity when examining the validity of administrative data relative to gold standard ASD assessment methods (Dodds et al. 2009). In Ontario, a person is eligible for disability income support if they are at least 18 years of age, an Ontario resident, be in financial need, and meet the program’s definition of having a disability. Disability is defined by a substantial mental or physical impairment that is continuous or recurrent, is expected to last 1 year or more, results in a substantial restriction in the ability to work, self-care, or take part in community life, and is verified by an approved health care professional.^1^ Applying this algorithm resulted in a group of 5095 individuals with ASD.

Those not identified as having ASD within the H-CARDD subset were placed in the other DD group, with one exception. To maintain greater specificity relative to sensitivity, we excluded individuals with only one physician billing code of ASD and no other database code of ASD (n = 398). This resulted in a group of n = 10,487 individuals with other DD. The non-DD group was comprised of a random sample of 20% of young adults from Ontario health administrative data, aged 18–24 years, who were not part of the H-CARDD cohort (n = 393,263).

The three groups were compared on demographics, clinical profiles, and health service use data for the 2009/2010 fiscal year. Demographic variables included age, sex (male, female); area of residence (urban or rural); and neighborhood income quintile, the latter two derived by linking 2006 Census data to individual residential postal code areas. The category “rural and small town” was designated for areas with a population of 10,000 or less. Income data were adjusted for household and community size, such that each community would be expected to have 20% of its population in each income quintile, and were ranked in five categories ranging from poorest (income quintile 1) to wealthiest (income quintile 5).

Health variables of interest included both physical and mental health conditions: diabetes, hypertension, asthma, psychiatric disorders, and substance-related and addictive disorders. Diabetes, hypertension, and asthma were chosen as past research comparing patients with ASD to the general population have often found higher rates in ASD. Validated algorithms for these conditions were used in the current study (Gershon et al. 2009; Hux et al. 2002; Quan et al. 2009). The look-back period for diabetes, hypertension, asthma, psychiatric disorders, and substance-related and addictive disorders was 2 years (2007–2009; Lin et al. 2016).

Health service use variables were family physician visits, pediatrician visits, specialist visits (gastroenterologist, neurologist, psychiatrist, respirologist, surgeon, and other specialist), any type of ED visits, ED visits specifically for psychiatric concerns, any type of hospital admission, and hospital admission specifically for psychiatric concerns. We calculated percentages of individuals with one or more of these health service variables in a 1-year period between April 1, 2009 and March 31, 2010.

### Statistical Analyses

Statistical analyses were performed with SAS version 9.2. Standardized differences were used to examine the differences in socio-demographic characteristics between groups. Standardized differences quantify the effect size for differences in proportions and are an extension of methods such as Cohen’s d (Austin 2009). The advantage of using standardized differences is that they are not influenced by sample sizes as are p-values, and are therefore more appropriate for large population-based studies (Mamdani et al. 2005). A standardized difference of at least 0.10 is viewed as clinically meaningful (Austin 2009). Clinical profiles and health service use of the three groups of young adults (with ASD, with other DD, and without DD) were compared by calculating unadjusted odds ratios (ORs) and adjusted ORs along with 95% confidence intervals, using logistic regression, whereby statistical significance was determined when confidence intervals did not overlap with 1. Adjusted ORs controlled for demographic characteristics (age, sex, rurality, and neighborhood income quintile). The study was approved by the institutional ethics review board at Sunnybrook Health Sciences Centre, the Centre for Addiction and Mental Health, and York University.

## Results

### Demographics

As shown in Table 2, the ASD group had a greater percentage of 18-year-olds, and fewer 24-year-olds, compared to the other DD group (standardized difference = 0.12 and 0.11, respectively). Similarly, the ASD group had a greater percentage of 18-year-olds (standardized difference = 0.19), and fewer 23- and 24-year-olds, than the non-DD group (standardized difference = 0.11 and 0.17, respectively). The ASD group was more likely to be male compared to those with other DD (standardized difference = 0.43), and to the non-DD group (standardized difference = 0.59). Those with ASD showed a similar pattern of distribution along income compared to the non-DD group, with a relatively even distribution at each quintile (e.g., lowest quintile: 21 versus 20.6%, standardized difference <0.03). In contrast, individuals with other DD were more likely to come from the lowest quintile (standardized difference = 0.15), and less likely to be in the highest quintile (standardized difference = 0.16) than those with ASD. Young adults with ASD were equally likely to come from rural regions compared to the comparison groups.

**Table 2:** Demographic characteristics between groups

	ASD N = 5095 n (%)	Other DD N = 10,487 n (%)	ASD versus other DD standardized difference^a^	Non-DD N = 393,263 n (%)	ASD versu non-DD standardized difference^a^
Sex (% male)	3954 (77.6)	6068 (57.9)	**0.43**	198,333 (50.4)	**0.59**
Ages					
18	1108 (21.8)	1792 (17.1)	**0.12**	57136 (14.5)	**0.19**
19	922 (18.1)	1762 (16.8)	0.03	57019 (14.5)	**0.10**
20	788 (15.5)	1522 (14.5)	0.03	55438 (14.1)	0.04
21	670 (13.2)	1421 (13.6)	0.01	54848 (14.0)	0.02
22	598 (11.7)	1392 (13.3)	0.05	55574 (14.1)	0.07
23	544 (10.7)	1277 (12.2)	0.05	56449 (14.4)	**0.11**
24	465 (9.1)	1321 (12.6)	**0.11**	56799 (14.4)	**0.17**
Income quintile					
Missing	53 (1.0)	157 (1.5)	0.04	7517 (1.9)	0.07
1	1071 (21.0)	2891 (27.6)	**0.15**	80,861 (20.6)	0.01
2	1015 (19.9)	2250 (21.5)	0.04	76,255 (19.4)	0.01
3	942 (18.5)	1883 (18.0)	0.01	75,187 (19.1)	0.02
4	960 (18.8)	1761 (16.8)	0.05	76,074 (19.3)	0.01
5	1054 (20.7)	1545 (14.7)	**0.16**	77,369 (19.7)	0.03
Rurality (% Rural)	589 (11.6)	1549 (14.8)	0.10	42,346 (10.8)	0.03

*ASD* autism spectrum disorder, *DD* developmental disability

^a^Standardized differences greater than 0.10 are clinically meaningful (indicated in bold) (Austin 2009)

### Clinical Profile Differences

Clinical profile differences are shown in Table 3. After controlling for demographics, compared to the non-DD group, those with ASD were more likely to have all the identified clinical health issues, including having 5.2 times greater odds of having at least one psychiatric diagnosis. However compared to those with other DD, young adults with ASD were *less* likely to have asthma, hypertension, and substance-related and addictive disorders, and *more* likely to have psychiatric diagnoses. In fact, the odds of an individual with ASD having at least one psychiatric diagnosis were 1.89 greater than those of their peers with other DD.

**Table 3:** Health and service use characteristics between groups

	% With outcome^a^			Unadjusted odd ratios (95% LCL–UCL)^b^		Adjusted odds ratios (95% LCL–UCL)^c^	
	ASD N = 5095	Other DD N = 10,487	Non-DD N = 393,263	ASD versus other DD	ASD versus non-DD	Adjusted ASD versus other DD	Adjusted ASD versus non-DD
Diabetes	1.67	1.99	0.65	0.83 (0.65–1.08)	2.58 (2.08–3.21)	0.88 (0.68–1.14)	2.78 (2.23–3.45)
Hypertension	1.67	2.62	0.83	0.63 (0.49–0.81)	2.02 (1.63–2.51)	0.62 (0.48–0.79)	2.03 (1.63–2.52)
Asthma	24.42	26.39	20.59	0.90 (0.83–0.97)	1.25 (1.17–1.33)	0.84 (0.77–0.90)	1.13 (1.06–1.21)
Psychiatric diagnosis	51.54	38.82	19.48	1.68 (1.57–1.79)	4.40 (4.16–4.65)	1.89 (1.77–2.03)	5.23 (4.95–5.33)
Substance-related and addictive disorder diagnosis	5.16	6.73	3.17	0.75 (0.65–0.87)	1.66 (1.47–1.88)	0.74 (0.64–0.86)	1.58 (1.39–1.79)
General practitioner visit	68.36	69.23	68.62	0.96 (0.89–1.03)	0.99 (0.93–1.05)	1.14 (1.06–1.23)	1.27 (1.20–1.35)
Pediatrician visit	7.91	6.29	2.15	1.28 (1.12–1.45)	3.90 (2.52–4.33)	1.30 (1.14–1.48)	4.00 (3.60–4.44)
Psychiatrist visit	22.61	11.29	2.43	2.30 (2.10–2.51)	11.71 (10.93–12.54)	2.34 (2.14–2.56)	12.03 (11.22–12.90)
Neurologist visit	5.77	5.57	1.01	1.04 (0.90–1.20)	6.03 (5.34–6.81)	1.14 (0.99–1.32_	6.90 (6.10–7.81)
Respirologist visit	0.29	0.62	0.21	0.47 (0.27–0.83)	1.39 (0.84–2.32)	0.49 (0.28–0.86)	1.48 (0.89-2.47)
Gastroenterologist visit	1.14	1.21	0.77	0.94 (0.69–1.28)	1.48 (1.14–1.92)	1.01 (0.74–1.38)	1.70 (1.31–2.21)
Surgical visit	10.72	17.03	13.76	0.58 (0.53–0.65)	0.75 (0.69–0.82)	0.73 (0.66–0.81)	1.02 (0.93-1.11)
Any emergency department visit	26.14	34.39	24.53	0.68 (0.63–0.73)	1.09 (1.02–1.16)	0.70 (0.65–0.76)	1.11 (1.04–1.18)
Emergency department visit for psychiatric reasons	8.04	6.71	1.80	1.21 (1.07–1.38)	4.77 (4.30–5.29)	1.23 (1.08–1.39)	4.58 (4.12 (5.09)
Any hospitalization	6.52	8.73	3.60	0.73 (0.64–0.83)	1.87 (1.67–2.09)	1.00 (0.88–1.14)	2.75 (2.45–3.08)
Psychiatric hospitalization	4.83	4.43	0.48	1.09 (0.93–1.29)	10.42 (9.09–11.93)	1.11 (0.95–1.31)	10.17 (8.86–11.67)

*ASD* autism spectrum disorder, *DD* developmental disability, *LCL* lower confidence limit, *HCL* higher confidence limit

^a^Expressed as a percentage of the group size

^b^Calculated using univariable logistic regression

^c^Calculated using multivariable logistic regression, controlling for demographic characteristics (age, sex, rurality, and neighbourhood income quintile)

### Health Service Use Differences

Health service use is also shown in Table 3. After controlling for demographics, young adults with ASD were more likely to have at least one visit with the family physician, pediatrician, psychiatrist, neurologist, gastroenterologist, and to visit the ED for psychiatric reasons, and be hospitalized overall and for psychiatric reasons, compared to the non-DD group. They were equally likely as the non-DD group to visit a respirologist or surgeon, and to visit the ED overall. Compared to young adults with other DD, the ASD group was *more* likely to have at least one visit to the family physician, pediatrician, and psychiatrist, and to visit the ED for psychiatric reasons; *less* likely to have a visit with a respirologist or surgeon and to have any type of ED visit; and *equally* likely to be hospitalized for any reason or psychiatric reasons, and to visit the neurologist or the gastroenterologist, compared to young adults with other DD.

## Discussion

This is the first population-based study of broad health status and health utilization in young adults with ASD compared to both young adults with other forms of DD and to those with no DD. The differences found between young adults with ASD and with no DD were in line with our hypotheses. Our findings echo other studies that have consistently shown that people with ASD (and other DD) have more medical and mental health issues compared to the general population and are more likely to use health, particularly hospital, based services (Croen et al. 2015; Cummings et al. 2016; Schlenz et al. 2015; Vohra et al. 2016). The current study largely stands out because of its identification of unique health concerns and health usage patterns of young adults with ASD relative to same age young adults with other DD. While the literature suggests that overall health use is higher in those with ASD, our findings suggest that this may be only when compared to the general population.

In our study, young adults with ASD had fewer or similar physical health issues and correspondingly less or similar health service use, compared to peers with other DD. Specifically, they were less likely to be diagnosed with either asthma or hypertension, and had similar rates of diabetes. Many of the genetic causes of intellectual impairment may have etiological repercussions for physical health conditions, explaining the relatively higher rate in the comparison group. For instance, young adults with intellectual disability without ASD have a higher likelihood of admission to hospital as a result of severe ear/nose/throat, respiratory, and genitourinary problems than do young adults with ASD (McDermott et al. 2015) and this is thought to relate to Down syndrome and Fragile X syndrome (Banjar 2012; Kidd et al. 2014; Kupferman et al. 2009; Hagerman et al. 2009; Shott 2006). It may also be that medical conditions not reflected in the current dataset may be higher in the ASD group, such as gastrointestinal problems (Croen et al. 2015).

This is not to say that health issues in those with ASD are unimportant, but rather that when training health care providers in internal medicine and emergency care, it is necessary that they understand there are health risks for all young adults with DD. A slightly broader healthcare training focus may assist many disability groups in addressing health needs, including patients with ASD. Further, even if some diseases are less common or similar at this specific age, how a patient with ASD may experiences healthcare may still differ. A reduced likelihood of health care visits may reflect less need or reflect more difficulties accessing or engaging with a service, because of the ASD-related stressors often present in the healthcare environment, or past negative experiences with the sector (Muskat et al. 2016). Certainly, in the case of ED visits for those with ASD, this has been suggested; parents are reluctant to return to an ED because the first visit can be highly stressful and traumatic for someone with ASD (Lunsky et al. 2015), and in our study, patients with ASD were less likely to visit the ED in general compared to other DD peers.

Even though medically young adults with ASD do not appear worse off than their peers with other DD, young adults with ASD were significantly more likely to have at least one psychiatric diagnosis, to visit a psychiatrist, and to have a psychiatric emergency department visit within the year. Mental health issues are common sources of stress and crisis for families living with ASD (Weiss et al. 2014), and likely account for the increased mental health service use found in our analysis. Findings here replicate patterns observed in non-population-based research. Clinical psychiatric interviews with parents of people with ASD and with ID consistently find that while both groups have higher rates of emotional and behavioral disorders compared to the general population, individuals with ASD present with even greater risk compared to those with ID without ASD (Bradley et al. 2004; Totsika et al. 2011). These studies have focused on the experience of children and youth with ASD. Our study reports on this occurrence in young adults, at a time when their health service providers change, along with disruptions in access to community services and alterations in daily routines as schooling comes to an end (Cheak-Zamora and Teti 2015; Shattuck et al. 2012). There is a clear need for mental health care supports for individuals with ASD in adulthood (Lake et al. 2014).

Unexpectedly, young adults with ASD did have a slightly higher likelihood of visiting the pediatrician that those with other DD, and certainly higher than the general population (OR of 1.3 and 4.0, respectively). One explanation is that the transition from pediatrician to family physician is more difficult for this group. In the US, it has been shown that adults with ASD have problems accessing family medicine (Nicolaidis et al. 2013). In a small scale Canadian study, a group of adults with ASD self-reported on their health service use over a year, with 1/3 of young adults with ASD not having a single visit with a primary care provider (Vogan et al. 2016). Given the higher rates of mental health problems, specific impairments in communication, and stress in unfamiliar situations (like hospital or ED) in adults with ASD, there is a need to offer holistic, proactive adult based care, similar to the medical home concept for children and adolescents (Walsh et al. 2017). The rise in prevalence of ASD in children over the last decade means that there will be even greater numbers of young adults with ASD entering the adult healthcare system in the near future, and tools are needed to help physicians and families navigate this transition so that all of them can access proactive care (McGonigle et al. 2014).

Strengths of this study include the size and generalizability of the cohort of young adults with ASD and other DD. By using health and social services administrative data to identify these young adults, we were able to achieve more complete ascertainment of disability status than is possible in clinical samples or using health data alone. Nevertheless, there are limitations to our study which should be acknowledged. Despite using linked health and social services data, some young adults with ASD and other DD may have been missed if they were not receiving income support for their disability and did not have a health care encounter with a relevant diagnostic code. Such misclassification would bias the results toward the null. Both ASD and other DD represent heterogeneous groups of conditions; although there could be variability in health and health service use according to the severity of the disability, we were not able to capture this information. Finally, we were unable to capture information on key medical issues known to be problematic for those with ASD (seizures and GI issues), and non-physician provided mental health services, including those delivered by psychologists or in community mental health centres.

## Conclusion

Based on the results, there is a need to plan health care for young adults with ASD in a way that is different not only from the general population, but also those with other DD. In particular, there is a considerable need for those with ASD in the area of mental health. As well as enhancing psychiatric services for people with ASD at this age, attention should also be paid to childhood and the transition years that lead up to young adulthood, to implement proactive care initiatives. Early identification of, and intervention for, psychiatric conditions for children and adolescents with ASD should be a priority, as should services that enhance mental well-being with the goal of preventing psychiatric illness.

## Footnotes

1
http://www.mcss.gov.on.ca/en/mcss/programs/social/odsp/income_support/IS_Eligibility.aspx.
